# The association of uric acid with mortality modifies at old age: data from the uric acid right for heart health (URRAH) study

**DOI:** 10.1097/HJH.0000000000003068

**Published:** 2021-12-22

**Authors:** Andrea Ungar, Giulia Rivasi, Mauro Di Bari, Agostino Virdis, Edoardo Casiglia, Stefano Masi, Alessandro Mengozzi, Carlo M. Barbagallo, Michele Bombelli, Bernardino Bruno, Arrigo F.G. Cicero, Massimo Cirillo, Pietro Cirillo, Giovambattista Desideri, Lanfranco D’elia, Claudio Ferri, Ferruccio Galletti, Loreto Gesualdo, Cristina Giannattasio, Guido Iaccarino, Michele Ciccarelli, Luciano Lippa, Francesca Mallamaci, Alessandro Maloberti, Alberto Mazza, Maria Lorenza Muiesan, Pietro Nazzaro, Paolo Palatini, Gianfranco Parati, Roberto Pontremoli, Fosca Quarti-Trevano, Marcello Rattazzi, Massimo Salvetti, Valérie Tikhonoff, Giuliano Tocci, Rosario Cianci, Paolo Verdecchia, Francesca Viazzi, Massimo Volpe, Guido Grassi, Claudio Borghi

**Affiliations:** a Department of Geriatric and Intensive Care Medicine, Careggi Hospital and University of Florence, Florence; b Department of Clinical and Experimental Medicine, University of Pisa, Pisa; c Department of Medicine, University of Padua, Padua; d Biomedical Department of Internal Medicine and Specialistics, University of Palermo, Palermo; e Clinica Medica, Department of Medicine and Surgery, University of Milano-Bicocca, Monza; f Department of Life, Health and Environmental Sciences, University of L’Aquila, L’Aquila; g Department of Medical and Surgical Science, Alma Mater Studiorum University of Bologna, Bologna; h Department of Public Health, “Federico II” University of Naples, Naples; i Nephrology, Dialysis and Transplantation Unit, Department of Emergency and Organ Transplantation, “Aldo Moro” University of Bari, Bari; j Department of Clinical Medicine and Surgery, “Federico II” University of Naples Medical School, Naples; k Cardiology IV, “A.De Gasperi's” Department, Niguarda Ca’ Granda Hospital, Milan; l School of Medicine and Surgery, Milano-Bicocca University, Milan; m Department of Advanced Biomedical Sciences, "Federico II" University of Naples, Naples; n Department of Medicine Surgery and Odontology, University of Salerno, Fisciano; o Italian Society of General Medicine (SIMG), Avezzano, L’Aquila; p CNR-IFC, Clinical Epidemiology of Renal Diseases and Hypertension, Reggio Cal Unit, Reggio Calabria; q Department of Internal Medicine, Santa Maria della Misericordia General Hospital, AULSS 5 Polesana, Rovigo; r Department of Clinical and Experimental Sciences, University of Brescia, Brescia; s Department of Medical Basic Sciences, Neurosciences and Sense Organs, University of Bari Medical School, Bari; t Istituto Auxologico Italiano, IRCCS, Department of Cardiovascular, Neural and Metabolic Sciences, San Luca Hospital, Milan; u Department of Medicine and Surgery, University of Milan-Bicocca, Milan; v Department of Internal Medicine, University of Genoa and Policlinico San Martino, Genoa; w Medicina Interna I, Ca’ Foncello University Hospital, Treviso; x Hypertension Unit, Division of Cardiology, Department of Clinical and Molecular Medicine, Faculty of Medicine and Psychology, University of Rome Sapienza, Sant’Andrea Hospital, Rome; y IRCCS Neuromed, Pozzilli; z Policlinico Umberto I, Sapienza University of Rome; aa Hospital S. Maria della Misericordia, Perugia, Italy

**Keywords:** cardiovascular prevention, cardiovascular risk, mortality, older adults, uric acid

## Abstract

**Objectives:**

In older individuals, the role of serum uric acid (SUA) as risk factor for mortality is debated. This study investigated the association of SUA with all-cause and cardiovascular (CV) mortality in older adults participating in the large multicentre observational uric acid right for heart health (URRAH) study.

**Methods:**

Eight thousand URRAH participants aged 65+ were included in the analysis. The predictive role of SUA was assessed using Cox regression models stratified according to the cut-off age of 75. SUA was tested as continuous and categorical variable (age-specific quartiles). The prognostic threshold of SUA for mortality was analysed using receiver operating characteristic curves.

**Results:**

Among participants aged 65–74, multivariate Cox regression analysis adjusted for CV risk factors and comorbidities identified an independent association of SUA with both all-cause mortality (hazard ratio [HR] 1.169, 95% confidence interval [CI] 1.107–1.235) and CV mortality (HR 1.146, 95% CI 1.064–1.235). The cut-off value of 4.8 mg/dl discriminated mortality status. In participants aged 75+, we observed a J-shaped relationship of SUA with all-cause and CV mortality, with risk increasing at extreme SUA levels.

**Conclusions:**

These results confirmed the predictive role of SUA for all-cause and CV mortality in older adults, while revealing considerable age-related differences. Mortality risk increased at higher SUA levels in participants aged 65–74, with a prognostic threshold of 4.8 mg/dl. The relationship between SUA and mortality was J-shaped in oldest participants. Large interventional studies are needed to clarify the benefits and possible risks of urate-lowering treatments in older adults.

## INTRODUCTION

Over recent years, serum uric acid (SUA) has gained increasing attention as a cardiovascular (CV) risk factor. A large number of epidemiological studies has reported an association of hyperuricemia with CV events and both CV and all-cause mortality [1–4] and experimental data have demonstrated that high SUA is associated with endothelial dysfunction, oxidative stress, increased platelet adhesiveness and inflammation [5–7]. Additionally, numerous studies have shown a link between hyperuricemia and other CV risk factors such as hypertension, obesity, dyslipidaemia and diabetes [8,9]. Finally, hyperuricemia has been proven to be an independent predictor of CV and renal diseases such as heart failure, stroke and chronic kidney disease [10–12]. Based on this clinical and physio-pathological evidence, SUA has been included among CV risk factors by the latest ESC/ESH Hypertension Guidelines [13].

Yet, it is worth considering that only few studies have explored the association between SUA and mortality in older adults [14–16]. Most evidence refers to middle-aged individuals or subset of the older population, for example, diabetic or hospitalized older patients, and available data on older individuals are inconsistent [17–20]. Additionally, some results suggest a J-shaped relationship between SUA and mortality at old age, with a slight risk increase in the presence of low SUA levels [14–17]. Therefore, whether the prognostic role of SUA observed in younger individuals can be extended to older people remains unclear.

It is well known that the prognostic relevance of traditional CV risk factors modifies at an advanced age, which hampers the generalisation of epidemiological data from younger to older individuals. In older adults, concomitant conditions such as multimorbidity, frailty and disability may confound the association between CV risk factors and adverse outcomes, leading to risk factor reversal [21]. In particular, observational data indicate that the negative prognostic role of hypertension, high cholesterol and obesity may be attenuated or even reverted in older persons [21]. As a consequence, thresholds for intervention and targets of treatment identified in younger individuals may not apply to older patients.

Recently, the Italian multicentre study “uric acid right for heart health” (URRAH) has confirmed the role of SUA as a predictor of all-cause and CV mortality in a large sample of normotensive and hypertensive individuals [22]. Additionally, it provided evidence that the threshold for increased mortality risk corresponds to SUA levels of 4.7 mg/dl (all-cause mortality) and 5.6 mg/dl (CV mortality), which would conversely be considered in the normal range [22].

As SUA levels are reported to increase with aging [23], a better understanding of the prognostic value of SUA in older individuals is merited, with the aim of clarifying the SUA threshold associated with excessive mortality risk. To this purpose, in the present study we investigated the association of SUA with all-cause and CV mortality focusing on older adults included in the URRAH study, in order to better explore the predictive role of SUA at an advanced age.

## METHODS

The URRAH Project is an Italian multicentre, retrospective, observational study assembling several cohorts of outpatients and individuals from the general population (age range 18–95 years), recruited all over Italy in the context of studies including SUA determination as part of the metabolic profile [24]. Data were merged to create a nationwide database where a standardized set of items was recorded, including demographics, anthropometric measures, metabolic parameters, smoking habit, systolic and diastolic arterial blood pressure, renal function, history of CV and renal disease, concomitant treatments and outcomes. More details on the study protocol have been provided elsewhere [24].

For the purposes of this analysis, we selected from the URRAH study population all participants aged 65 or older for whom follow-up data were available. The selected age cut-off was consistent with previous studies [16,19,25]. In addition, age-stratified analyses were also performed using a predefined cut-off of 75, to investigate possible age-related changes in the prognostic value of SUA.

### Outcomes and other measures

Study outcomes included mortality for any cause (all-cause mortality) and fatal events due to acute myocardial infarction, heart failure, or stroke (CV mortality) [23]. Information concerning vital status was obtained from hospital records or death certificates. Follow-up data were censored at the time of the last visit or, for participants lost during follow-up, at the last date they were known to be alive.

Diabetes was defined in presence of antidiabetic therapy, fasting plasma glucose ≥126 mg/dl, or haemoglobin A1c ≥48 mmol/mol. Hypertension was defined in presence of antihypertensive treatment or at least two blood pressure recordings >140/90 mmHg. Chronic kidney disease was defined as glomerular filtration rate <60 ml/min, which in turn was estimated using the Chronic Kidney Disease Epidemiology Collaboration equation based on serum creatinine [26]. Additional information on variable assessment have been outlined elsewhere [23].

### Ethics

The URRAH project was performed according to the Declaration of Helsinki for Human Research. Data were collected in previous studies, whose protocols had been approved by the local Ethics Committee of each participating Centre. Additionally, approval was sought from the Ethical Committee of the Coordinating Centre at the Division of Internal Medicine of the University of Bologna (No. 77/2018/Oss/AOUBo). A written informed consent was obtained from all of the participants at enrolment. All data are available from the URRAH Steering Committee upon request.

### Statistical analysis

Results are presented as mean and standard deviation for normally distributed interval variables, as median and interquartile range for non-normally distributed variables and absolute frequencies with percentages for categorical variables. The independent samples *t*-test (parametric) or the Mann–Whitney *U* test (non-parametric) were used as appropriate for comparisons of interval variables. For categorical variables, differences between groups were tested using the *χ*^2^ test.

Mortality prediction was assessed using SUA as a continuous variable (in mg/dl) in Cox proportional hazards regression models adjusted for age, sex, and other variables (CV risk factors, renal function, heart failure, diuretic use) with potential influence on the outcome of interest. Associations are presented as hazard ratio (HR) with 95% confidence interval (CI). The predictive role of SUA for mortality was also examined in age-stratified analyses, using a predefined cut-off of 75.

In the presence of an independent association with mortality, the receiver operating characteristic (ROC) curves method was used to define the prognostic cut-off of SUA levels, which was identified as the SUA value corresponding to the optimal combination of sensitivity and 1 − specificity. The cut-off SUA value was then included as independent variable in a multivariate Cox regression model having mortality as dependent variable and the above-described variables as confounders. Additionally, the same cut-off value was used to generate Kaplan–Meier survival curves.

Since all-cause and CV mortality rates did not vary linearly with SUA levels in the two age subgroups, the association with mortality was also analysed after categorizing SUA into age-specific quartiles, which were included in a multivariate Cox regression model with the lowest risk quartile as the reference category.

Statistical significance was set at a *P* value <0.05. Analyses were performed using SPSS Statistics package, version 26 (IBM Corp, Armonk, New York, USA).

## RESULTS

Table 1 illustrates the characteristics of older URRAH study participants (*N* = 8000, 45% female), stratified by age subgroups. During a median follow-up of 11.0 years (IQR 6.0–12.6), all-cause and CV mortality were 31.3 and 16.4%, respectively, with significantly higher figures in the older subgroup (all-cause mortality: 46.9 vs. 23.6%; CV mortality: 27.2 vs. 11.0%; *P* < 0.001 for both).

**TABLE 1:** Baseline characteristics of the study sample stratified by age subgroups

	Study sample (*n* = 8000)	Age 65–74 (*n* = 5335)	Age 75+ (*n* = 2665)	*P*
Age (years), mean ± SD	72.3 ± 5.6	69.5 ± 2.7	79.4 ± 3.9	–
Female, *n* (%)	3575 (44.7)	2822 (52.9)	1603 (60.2)	<0.001
Uric acid (mg/dl), mean ± SD	5.3 ± 1.4	5.2 ± 1.4	5.4 ± 1.5	<0.001
Hypertension (*n* = 6612), *n* (%)	3366 (50.9)	2134 (48.2)	1232 (56.3)	<0.001
Diabetes, *n* (%)	1435 (17.9)	876 (16.5)	559 (21.0)	<0.001
Smoking, *n* (%)	1310 (16.4)	990 (18.6)	320 (12.0)	<0.001
Chronic kidney disease, *n* (%)	1862 (23.3)	1168 (22.0)	694 (26.1)	<0.001
Gout (*n* = 6429), *n* (%)	93 (1.4)	52 (1.2)	41 (1.8)	0.068
Allopurinol (*n* = 6338), *n* (%)	129 (2.0)	69 (1.7)	60 (2.7)	0.005
Heart failure (*n* = 7408), *n* (%)	2196 (29.6)	1235 (25.6)	961 (37.3)	<0.001
Creatinine (mg/dl), mean ± SD	0.96 ± 0.3	0.94 ± 0.28	0.99 ± 0.34	<0.001
BMI (kg/m^2^), mean ± SD	27.1 ± 4.3	27.3 ± 4.3	26.7 ± 4.3	<0.001
SBP (mmHg), mean ± SD (*n* = 7778)	154.6 ± 24.1	153.5 ± 23.7	156.8 ± 24.8	<0.001
DBP (mmHg), mean ± SD (*n* = 7778)	85.4 ± 12.6	86.4 ± 12.4	83.5 ± 12.7	<0.001
Diuretic therapy, *n* (%)	1484 (18.6)	951 (18.9)	533 (20.2)	0.175
Hydrochlorothiazide (*n* = 2328), *n* (%)	345 (14.8)	196 (14.0)	149 (16.1)	0.161
Loop diuretics (*n* = 5601), *n* (%)	425 (7.6)	203 (5.9)	222 (10.2)	<0.001

BMI, body mass index; CV, cardiovascular; DBP, diastolic blood pressure; IQR, interquartile range; SBP, systolic blood pressure; SD, standard deviation.

### Association of serum uric acid as a continuous variable with all-cause mortality

In univariate Cox regression analysis, SUA was associated with an increased risk of all-cause mortality (HR 1.141 per 1 mg/dl increase of SUA, [95% CI 1.111–1.171]; *P* < 0.001). This association remained significant in a multivariate Cox regression model adjusted for age, sex, other CV risk factors, heart failure, chronic kidney disease, and diuretic use (Supplementary Table 1). Hypertension, diabetes and heart failure also contributed to mortality, while total cholesterol levels showed an inverse association with the outcome.

The age-stratified Cox regression analysis confirmed an independent association between SUA and all-cause mortality in younger participants (65–74 years). In participants aged 75 or older, SUA levels were not associated with all-cause mortality when adjusting for confounders (Table 2). Total cholesterol was inversely related to all-cause mortality in the older age subgroup, but not in the younger one (Table 2).

**TABLE 2:** Age-stratified multivariate Cox regression analyses for all-cause mortality using serum uric acid as a continuous independent variable

	Hazard ratio	95% CI (lower bound)	95% CI (upper bound)	*P*
Age 65–74 years				
Uric acid (mg/dl)^a^	1.185	1.142	1.230	<0.001
Uric acid (mg/dl)	1.169	1.107	1.235	<0.001
Male sex	1.944	1.662	2.273	<0.001
Hypertension	1.085	0.924	1.273	0.321
Alcohol use	2.162	1.663	2.810	<0.001
Creatinine (mg/dl)	1.228	0.985	1.531	0.068
Total cholesterol (mg/dl)	0.999	0.997	1.001	0.229
Diuretic use	0.920	0.728	1.162	0.483
Diabetes	1.726	1.450	2.054	<0.001
Chronic kidney disease	0.882	0.733	1.062	0.185
Heart failure	2.096	1.788	2.457	<0.001
Age 75+ years				
Uric acid (mg/dl)^a^	1.064	1.024	1.105	0.002
Uric acid (mg/dl)	1.009	0.570	1.064	0.730
Male sex	1.340	1.140	1.574	<0.001
Hypertension	1.184	1.016	1.380	0.030
Alcohol use	2.352	1.811	3.054	<0.001
Creatinine (mg/dl)	0.964	0.768	1.210	0.752
Total cholesterol (mg/dl)	0.997	0.995	0.999	0.001
Diuretic use	0.619	0.493	0.777	<0.001
Diabetes	1.591	1.332	1.900	<0.001
Chronic kidney disease	0.899	0.735	1.100	0.301
Heart failure	2.133	1.807	2.518	<0.001

a Unadjusted.

**FIGURE 1:**
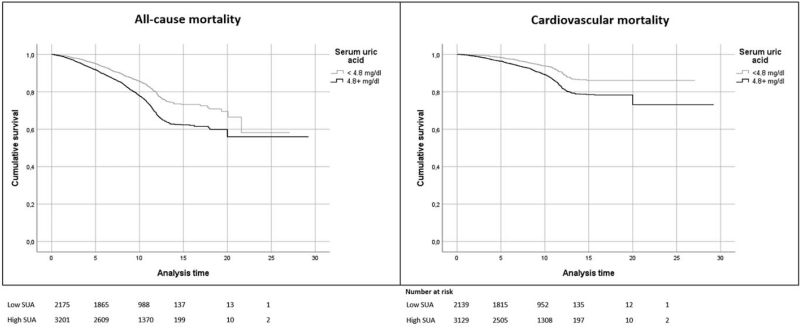
Kaplan–Meier survival estimates for all-cause and cardiovascular mortality in participants aged 65–74 years. Trends of individuals having serum uric acid 4.8 mg/dl or higher (solid black line) and lower than 4.8 mg/dl (light gray line) are compared. *P* < 0.001 for all, log-rank test. Analysis time is expressed in years.

In participants aged 65–74 years, the ROC curve analysis showed that the optimal cut-off SUA value for all-cause mortality was 4.8 mg/dl (sensitivity 68%, specificity 43%; AUC 0.573). Kaplan–Meier curves based on the identified cut-off showed that SUA levels ≥4.8 mg/dl were associated with an increased risk of all-cause mortality (Fig. 1, left panel). The cut-off value of SUA for all-cause mortality in participants aged 65–74 years (4.8 mg/dl) was also retained in a multivariate Cox analysis adjusted for sex, hypertension, diabetes, chronic kidney disease, heart failure and diuretic use (HR 1.452 [95% CI 1.258–1.676], Supplementary Table 2).

### Association of serum uric acid quartiles with all-cause mortality

When stratifying according to age-specific SUA quartiles (Supplementary Table 3), all-cause mortality progressively increased with SUA concentrations in participants aged 65–74 years (*P* for trend < 0.001). Conversely, all-cause mortality rates showed a J-shaped distribution in participants aged 75 or older (Supplementary Table 3), with the lowest value in the second quartile of SUA distribution (4.30–5.19 mg/dl).

**FIGURE 2:**
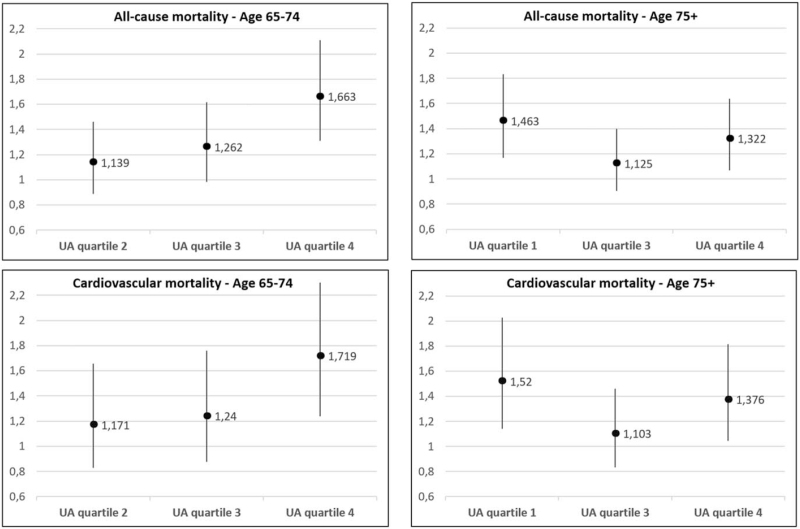
Hazard ratios and 95% confidence intervals of uric acid age-specific quartiles for all-cause and cardiovascular mortality. Multivariable Cox analysis adjusted for sex, hypertension, creatinine (mg/dl), total cholesterol (mg/dl), alcohol use, diuretic use, diabetes, chronic kidney disease, heart failure. Division points for quartiles: 4.2, 5.1 and 6.0 mg/dl in participants aged 65–74 years, with the lowest quartile as reference; 4.3, 5.2 and 6.2 mg/dl in participants aged 75+ years, with the second quartile as reference. UA, uric acid.

SUA quartiles were then included in age-stratified multivariate Cox analyses considering the lowest mortality quartile as the reference. In patients aged 65–74 years, SUA levels in the highest quartile were associated with a greater risk of all-cause mortality (HR 1.663, 95% CI 1.311–2.110) as compared to the lowest SUA quartile (Fig. 2, left panel). Among participants aged 75 or older, mortality risk was significantly greater for the lowest and the highest quartile in relation to the second (Fig. 2, right panel).

### Association of serum uric acid as continuous variable with cardiovascular mortality

In univariate Cox regression analysis, SUA was associated with an increased risk of CV mortality (HR 1.167 per 1 mg/dl increase of SUA, [95% CI 1.125–1.211]; *P* < 0.001). This association was confirmed in a multivariate Cox model adjusted for age, sex, other CV risk factors, heart failure, chronic kidney disease, alcohol and diuretic use (Supplementary Table 4). Hypertension, diabetes, heart failure, and chronic kidney disease also contributed to CV mortality.

The age-stratified Cox regression analysis confirmed an independent association between SUA and CV mortality in participants aged 65–74 years, but not in older individuals (Table 3). In participants aged 65–74 years, the ROC curve analysis showed that the optimal cut-off SUA value to discriminate CV mortality was 4.8 mg/dl (sensibility 71%, specificity 42%; AUC 0.587, *P* < 0.001). At the Kaplan–Meier curves, SUA levels ≥4.8 mg/dl were associated with an increased risk of CV mortality in the younger subgroup (Fig. 1, right panel). This cut-off value was also retained in a multivariate Cox analysis adjusted for sex, hypertension, diabetes, chronic kidney disease, heart failure and diuretic use (HR 1.463 [95% CI 1.198–1.787], Supplementary Table 5).

**TABLE 3:** Age-stratified multivariate Cox analysis for cardiovascular mortality using serum uric acid as a continuous independent variable

	HR	95% CI (lower bound)	95% CI (upper bound)	*P*
Age 65–74 years				
Uric acid (mg/dl)^a^	1.236	1.171	1.305	<0.001
Uric acid (mg/dl)	1.146	1.064	1.235	<0.001
Male sex	1.857	1.497	2.305	<0.001
Hypertension	1.269	1.016	1.584	0.035
Alcohol use	2.419	1.679	3.484	<0.001
Creatinine (mg/dl)	1.303	1.029	1.649	0.028
Total cholesterol (mg/dl)	1.000	0.998	1.003	0.802
Diuretic use	0.950	0.701	1.288	0.740
Diabetes	1.982	1.567	2.506	<0.001
Chronic kidney disease	1.318	1.034	1.680	0.026
Heart failure	2.461	1.973	3.069	<0.001
Age 75+ years				
Uric acid (mg/dl)^a^	1.074	1.022	1.129	0.005
Uric acid (mg/dl)	1.008	0.941	1.080	0.823
Male sex	1.291	1.046	1.594	0.017
Hypertension	1.375	1.130	1.673	0.001
Alcohol use	2.347	1.703	3.236	<0.001
Creatinine (mg/dl)	0.740	0.484	1.130	0.163
Total cholesterol (mg/dl)	0.998	0.995	1.000	0.062
Diuretic use	0.607	0.458	0.805	0.001
Diabetes	1.770	1.417	2.212	<0.001
Chronic kidney disease	1.152	0.890	1.492	0.282
Heart failure	2.158	1.740	2.675	<0.001

a Unadjusted.

### Association of serum uric acid quartiles with cardiovascular mortality

When stratifying according to age-specific SUA quartiles (Supplementary Table 3), CV mortality progressively increased across quartiles in participants aged 65–74 years (*P* < 0.001). Similarly to all-cause mortality, CV mortality showed a J-shaped distribution in participants aged 75 or older, with the lowest level in the second quartile of SUA distribution (4.20–5.09 mg/dl). In participants aged 65–74 years, SUA levels in the highest quartile predicted an increased risk of CV mortality (HR 1.719, 95% CI 1.237–2.389) as compared to the lowest quartile (Fig. 2, left panel). Among participants aged 75 or older, mortality risk was significantly greater for the lowest and the highest quartile as compared to the second (Fig. 2, right panel).

## DISCUSSION

The present analysis from the large URRAH study database investigated the prognostic value of SUA in older adults and confirmed its role as a risk factor for both all-cause and CV mortality, while revealing substantial age-related differences. Among participants aged 65–74 years, higher SUA levels were associated with greater mortality risk, starting from the cut-off value of 4.8 mg/dl. Conversely, in participants aged 75 or older, we observed a J-shaped relationship between SUA and mortality, with a significant risk increase in individuals with both low and high SUA levels.

To date, the association between SUA and mortality has been scarcely investigated in older patients and available data are inconsistent. Some studies revealed an increased risk of all-cause and CV mortality in older adults presenting with high SUA levels [14–15,20,27]. In a study by Wu *et al.*, high SUA was identified as a predictor all-cause and CV mortality in community dwelling older adults, independently of other traditional CV risk factors [16]. In the Cardiovascular Study in the Elderly (CASTEL), SUA was independently associated with coronary mortality in diabetic older patients [17]. Finally, in recent studies SUA predicted in-hospital and long-term all-cause mortality in older hospitalized patients [18,28]. By contrast, other authors do not confirm these associations, implying that the prognostic relevance of SUA does not apply to older individuals [19]. Finally, although some studies agree on the predictive value of SUA at an advanced age, data on the prognostic serum value are conflicting. The Established Populations for Epidemiologic Studies of the Elderly, Iowa (Iowa-EPESE) and the Third National Health and Nutritional Examination Survey (NHANES III) indicate a 38% risk increase in CV mortality in participants aged 70 or older presenting with SUA > 7 mg/dl [15]. In the InCHIANTI Study, the risk of CV mortality significantly increased for SUA levels above 4.3 mg/dl [25]. Moreover, several studies describe a J- or a U-shaped relationship between SUA and mortality, with risk elevation in older adults with extreme SUA levels [15–17,25,29]. Indeed, Tseng *et al.* reported a higher risk of all-cause mortality in older adults with SUA <4 mg/dl and >8 mg/dl. Comparable results were observed for CV mortality, with risk increase at SUA <4 mg/dl and >7 mg/dl [30].

Our data from a sample of 8000 URRAH participants aged 65 or older provided evidence supporting the predictive value of SUA for all-cause and CV mortality in older individuals, but also confirmed that this association is complex and strongly age-related. In the younger subgroup of our study sample (65–74 years) both all-cause and CV mortality increased at higher SUA levels, with the same cut-off value of 4.8 mg/dl discriminating mortality status. This prognostic threshold was similar to the one reported in the overall URRAH database for all-cause mortality (4.7 mg/dl), but slightly lower than the one reported for CV mortality (5.6 mg/dl) [23]. In the older subgroup of our sample (participants aged 75 or older), SUA as a continuous variable was not associated with all-cause and CV mortality when adjusting for confounders including comorbidities and other CV risk factors. Yet, a J-shaped association was observed between SUA levels and mortality risk when participants were grouped according to SUA quartiles, in agreement with previous studies. We may thus observe that the prognostic role of SUA significantly modifies with age, similarly to what reported for other CV risk factors. Indeed, it is well known that the association of many CV risk factors with adverse events attenuates or even inverts at advanced age, thus resulting in the phenomenon of “risk factor reversal” (or “risk factor paradoxes”) [21]. In older adults, body mass index values in the high-normal to overweight ranges seem to carry a lower risk of adverse outcomes than being low-normal or underweight, a phenomenon which is referred to as “obesity paradox” [21]. Similarly, hypertension appears to be a less prominent risk factor in older age. Aging is associated with a progressive attenuation in the protective effects of lower blood pressure and some degrees of hypertension are suggested to have survival advantage or at least no survival disadvantage at advanced age, particularly in the presence of frailty and cognitive impairment [31]. Finally, the role of cholesterol as a risk factor in the geriatric population is controversial. Numerous epidemiological studies support the presence of the “cholesterol paradox” in older adults [21], suggesting that increasing cholesterol levels provide survival advantage over lower levels, mainly due to lower mortality from cancer and infections [32–34]. In accordance with this evidence, our study described an inverse association between total cholesterol and all-cause mortality, particularly in individuals aged 75 or older.

As regards SUA, the present analysis corroborates its role as a risk factor for mortality in the geriatric population, although its predictive role seems to be attenuated in older individuals. In addition, our study also provides evidence for a negative prognostic value of very low SUA levels in older individuals, in parallel with what reported for other CV risk factors. Although the possible reasons for this phenomenon remain unclear, some authors suggest reverse causation as a possible explanation. Indeed, it is known that malnutrition may contribute to low SUA levels, which are considered as surrogate of inadequate protein and caloric intake. Therefore, the decrease of SUA in individuals with malnutrition may at least partly explain low-SUA related mortality in older adults [30,35]. Low SUA levels would thus represent a marker of poor health status, as previously reported for low cholesterol [36].

Given the known antioxidant properties of SUA [37], we may also hypothesize that very low SUA might determine a reduced ability to counteract oxidative stress and damage in very old adults. Finally, frailty and comorbidities may confound the association between SUA and mortality at advanced age. Indeed, in the geriatric population life expectancy may be shorter due to competing conditions, which play a more relevant role in patients’ prognosis as compared to CV risk factors. Indeed, time-until onset of adverse consequences of CV risk factors might exceed the life expectancy, thus resulting in their blunted prognostic impact.

Prevalence of heart failure and diuretic therapy were considerable in our study sample and may have contributed to higher SUA levels. Recent data from the URRAH study have demonstrated that diuretic-related hyperuricemia carries a similar risk of cardiovascular events and all-cause mortality as compared to hyperuricemia occurring in patients not receiving diuretic therapy [38]. Therefore, diuretics do not seem to modify significantly the prognostic relevance of SUA levels, particularly when multiple potential confounding factors are taken into consideration [38].

As the association between SUA levels and mortality significantly modifies with age, future research should investigate the prognostic impact of urate-lowering treatments in the geriatric population, with particular reference to very old individuals.

### Limitations

Our results must be interpreted in the context of some study limitations. First, the URRAH project dataset did not include variables with significant prognostic impact in the geriatric population, for example, frailty, functional level and nutritional status. Therefore, we were unable to assess whether these variables could influence the relationship between SUA and mortality. In particular, we were unable to investigate the prognostic value of malnutrition and sarcopenia, which may contribute to lower serum uric acid levels thus confounding the association between uric acid and mortality at old age. Moreover, low muscle mass is common in older adults and may lead to very low serum creatinine values. As a result, renal function may be overestimated when eGFR is calculated based on creatinine levels. Cystatin C would likely be a more appropriate marker of renal function in this age group, but unfortunately cystatin C levels were not available in the URRAH database. The URRAH study had a retrospective design and the analysis was based on a single SUA measurement. Consequently, the effect of changes in SUA levels during follow-up were not investigated. Finally, SUA levels and mortality risk were relatively low in the URRAH database [23]. Therefore, our results cannot be extrapolated to populations with higher SUA levels or with a different CV risk profile. Finally, the URRAH study focused on cardiovascular risk, while no details were available on non-cardiovascular causes of mortality, which could have provided further insights into the prognostic role of uric acid.

In conclusion, higher SUA levels were independently associated with an increased risk of all-cause and CV mortality in URRAH study participants aged 65–74 years, with a prognostic cut-off value of 4.8 mg/dl. In participants aged 75 or older, the association between SUA and mortality was J-shaped, with a significant risk increase at both low and high SUA levels. Large interventional studies are needed to clarify the benefits and possible risks of urate-lowering treatments in older adults.

Source of Funding: This research has been conducted with an unrestricted grant from the Fondazione of the Italian Society of Hypertension (Grant: MIOL).

## ACKNOWLEDGEMENTS

### Conflicts of interest

There are no conflicts of interest.
